# Long-Term Management of Advanced Basal Cell Carcinoma: Current Challenges and Future Perspectives

**DOI:** 10.3390/cancers14194547

**Published:** 2022-09-20

**Authors:** Markus V. Heppt, Christoffer Gebhardt, Jessica C. Hassel, Mareike Alter, Ralf Gutzmer, Ulrike Leiter, Carola Berking

**Affiliations:** 1 Department of Dermatology, Uniklinikum Erlangen, Friedrich-Alexander-University Erlangen-Nürnberg, 91054 Erlangen, Germany; 2 Comprehensive Cancer Center Erlangen-European Metropolitan Area of Nuremberg (CC ER-EMN), 91054 Erlangen, Germany; 3 Department of Dermatology and Venereology, University Medical Center Hamburg-Eppendorf (UKE), 20246 Hamburg, Germany; 4 Department of Dermatology and National Center for Tumor Diseases (NCT), University Hospital Heidelberg, 69120 Erlangen, Germany; 5 Department of Dermatology, Johannes Wesling Medical Center, Ruhr University Bochum Campus Minden, 32423 Minden, Germany; 6 Department of Dermatology, Eberhard-Karls-University Tuebingen, 72076 Tuebingen, Germany

**Keywords:** basal cell carcinoma, Hedgehog pathway inhibitors, immunotherapy, programmed cell death protein 1 inhibitor

## Abstract

**Simple Summary:**

Local therapies are no longer an option for locally advanced basal cell carcinoma. Abnormal activation of the hedgehog signaling pathway leads to uncontrolled tumor growth. Hedgehog pathway inhibitors are an effective treatment option for this kind of tumor. However, treatment-related toxicity under long-term treatment may lead to limitations in quality of life, and thus to therapy interruption or even discontinuation. This review summarizes pertinent treatment adjustments and novel therapeutic strategies for effective treatment of locally advanced basal cell carcinoma.

**Abstract:**

The first-line therapy for locally advanced basal cell carcinoma (laBCC) is Hedgehog pathway inhibitors (HHIs), as they achieve good efficacy and duration of response. However, toxicity in the course of long-term treatment may lead to a decrease in the quality of life, and consequently to interruption or even discontinuation of therapy. As HHI therapy is a balancing act between effectiveness, adverse events, quality of life, and adherence, numerous successful treatment strategies have evolved, such as dose reduction and dose interruptions with on-off treatment schedules or interruptions with re-challenge after progression. As a small percentage of patients show primary or acquired resistance to HHIs, the inhibition of programmed cell death protein 1 (PD-1) has been approved as a second-line therapy, which may also be accompanied by immune-related toxicities and non-response. Thus, optimization of current treatment schedules, novel agents, and combination strategies are urgently needed for laBCC. Here, we narratively model the treatment sequence for patients with laBCC and summarize the current state of approved treatment regimens and therapeutic strategies to optimize the long-term management of laBCC.

## 1. Introduction

Basal cell carcinoma (BCC) is the most commonly diagnosed human cancer in the Caucasian population [1]. Age and ultraviolet (UV) light exposure are among the most important risk factors for the development of BCC [2]. Patients with the rare heritable basal cell nevus syndrome (BCNS, also known as Gorlin–Goltz syndrome) have a genetic predisposition to develop BCCs [1]. Most cases of BCC can be treated surgically or less frequently with radiotherapy, topical, or physical treatments [3,4]. However, in some cases the disease may progress to advanced BCC (aBCC), including locally advanced BCC (laBCC) and metastatic BCC (mBCC), for which local therapies are no longer an option [5].

**Figure 1:**
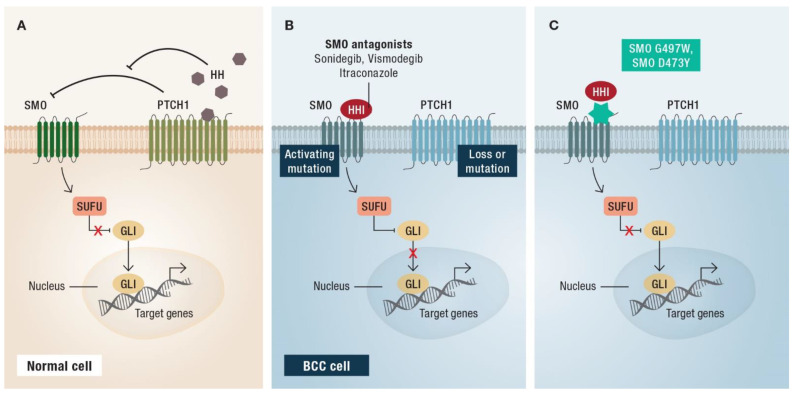
Schematic representation of the Hedgehog (HH) signaling pathway. (**A**) Binding of the extracellular HH ligand to the Patched 1 (PTCH1) receptor releases Smoothened (SMO) from inhibition. SMO starts an intracellular signaling cascade including Suppressor of Fused (SUFU), which results in activation of the transcription factors of the GLI family; (**B**) loss-of-function mutations in *PTCH1* or activating mutations in *SMO* activate the HH signaling pathway in basal cell carcinoma (BCC) cells. This process can be inhibited by HH pathway inhibitors (HHI); (**C**) intrinsic resistance as caused by SMO G497W mutation, or acquired resistance as caused by SMO D473Y mutation impede entry of the drug into the binding site. Modified according to [1].

Aberrant activation of the Hedgehog (HH) pathway (Figure 1A) is a key pathophysiological event in the development of BCC. In 80–90% of tumors, loss-of-function mutations in the gene encoding the twelve-pass transmembrane receptor Patched 1 (*PTCH1*) can be identified (Figure 1B). Approximately 10% of tumors arise due to activating mutations in the gene Smoothened (*SMO*; Figure 1B) [6,7,8,9]. The HH signaling pathway results in the activation of transcription factors of the GLI family (Figure 1A) and controls cell differentiation and proliferation in keratinocytes [1]. The HH pathway therefore maintains cutaneous stem cell populations and regulates sebaceous gland and hair follicle development. Aberrant activation results in uncontrolled tumor growth and survival. Since the HH signaling pathway is the most important factor in the pathogenesis and progression of BCC, it is an important therapeutic target.

HH pathway inhibitors (HHIs), such as vismodegib and sonidegib, are small molecule antagonists that bind and inhibit SMO; thereby preventing downstream signaling (Figure 1B) [10,11]. Vismodegib has been approved as first-in-class HHI for the treatment of adults with laBCC or mBCC that is unsuitable for surgery or radiotherapy [12]. More recently, sonidegib was approved as a second HHI for laBCC [13].

Although these drugs are effective and relatively well tolerated, treatment-related toxic effects under long-term treatment can lead to a decrease in patients’ quality of life [14]. The most commonly observed adverse events (AEs) in HHI-treated patients include muscle spasms, ageusia/dysgeusia, alopecia, weight loss, and asthenia (fatigue) [15]. This may result in early treatment interruption and even discontinuation and may thus impact the clinical outcome. For the clinician, this means a balancing act between effectiveness, AEs, quality of life, and adherence. There is, therefore, a high unmet need for the treatment of BCC patients requiring long-term treatment. Since there are only a few large studies and no head-to-head studies in the field of laBCC therapy, real-world data and case series provide valuable insights into therapeutic options. Numerous publications of case series and real-world data with HHI suggest many different schemes of dose reduction [16,17,18,19] or treatment interruption [20,21,22,23] in order to increase patient adherence to treatment. Immunotherapy with programmed cell death protein 1 (PD-1) inhibitors has recently become available as second-line therapy. As this approach leads to a response in approximately 30% of patients [24], further strategies are urgently needed to allow long-term management of the disease even after second-line treatment. Here, we summarize the current state of treatment regimens and therapeutic strategies for aBCC.

## 2. First-Line Therapy: Hedgehog Inhibitors

The HHI sonidegib (200 mg) was approved for laBCC based on results of the BOLT trial (NCT01327053), a double-blind, two arm (200 mg vs. 800 mg sonidegib once daily [QD]) phase II study that evaluated the long-term efficacy and safety of sonidegib for laBCC (*n* = 194) and mBCC (*n* = 36) [25]. Vismodegib (150 mg), another HHI, was approved based on the ERIVANCE trial (NCT00833417), a single-arm, multicenter, 2-cohort phase II study that evaluated the efficacy and safety of vismodegib in patients with aBCC (laBCC *n* = 71, mBCC *n* = 33) [26]. The efficacy data of sonidegib and vismodegib from the pivotal studies showed response rates that ranged from 47% to 60% based on comparable evaluation methods (response evaluation criteria in solid tumors, RECIST; Table 1) [25,26]. Complete response (CR) was observed in slightly more than 20% for both drugs [25,26,27]. The median duration of response (DOR) by central review was 26.1 months for sonidegib [25,28] and 9.5 months for vismodegib [26]. Sonidegib showed a high disease control rate (DCR: CR + partial response [PR] + stable disease [SD]) of 92.3% [25,28].

After approval, subsequent studies investigated the efficacy, safety, and utilization of these two HHIs for the treatment of laBCC in real-world clinical practice. The non-interventional study NIELS assessed the effectiveness and safety of vismodegib for the treatment of laBCC under real-world conditions in Germany [29]. A total of 66 patients were observed at 26 German centers. Complete discontinuation of treatment due to adverse events only occurred in one patient, but 36% of the patients interrupted treatment because of side effects with a median interruption of 7.6 months before re-challenge. This approach of AE management, with interruptions and re-challenge, still led to an objective response rate (ORR) of 74.2% and a median DOR and median progression-free survival (mPFS) of 15.9 months and 19.1 months, respectively. After 42 months, 60% of the patients with an initial CR had a recurrence, which is probably because of the interruption of treatment after the CR until progression.

To evaluate the efficacy of sonidegib under real-world conditions, 21 patients were studied in the retrospective, single-center observational study PaSoS [30]. An ORR of 81% was achieved. Nine patients underwent surgery after discontinuation of sonidegib. None of them experienced disease progression. The median time to first tumor response was 2.3 months. In this study, it was reported for the first time that the time to maximal response was 3.2 months, suggesting that the patients’ response peaked just slightly after their initial response. Leow and Teh recently published a case series of 10 patients with laBCC who were treated with 200 mg sonidegib QD [31]. Eight patients had no residual BCC following sonidegib treatment and two patients experienced sufficient tumor size reduction to allow surgical removal of the tumor. Villani et al. responded to this study by reporting their real-life experience of treating 36 laBCC patients with sonidegib (200 mg daily) [32]. To reduce the degree of AEs, 10 patients received an alternative dosing regimen (200 mg every other day; see Section 3.2). In total, 47.2% of patients achieved CR, 38.9% had >50% clinical reduction of the laBCC, and 13.9% showed no treatment response (<50% of tumor size reduction).

Overall, real-life data have confirmed the efficacy and tolerability profile of HHIs in laBCC. Remarkably, response rates in real-world studies were even higher than those in pivotal studies. One possible explanation for these differences could be a discrepancy between the populations studied. In the pivotal study, 89% of patients had surgery prior to vismodegib treatment [33]. By contrast, 22.7% of patients in the NIELS study were treatment-naïve for BCC therapy [29]. In addition, in pivotal studies, strict RECIST evaluation criteria are used to measure effectiveness. Those criteria require photography, magnetic resonance imaging, and biopsies, and investigator reviews are also centrally reviewed.

Although AEs for both HHIs are primarily low-grade (<2) [34], discontinuation rates are higher in pivotal studies than in real-world practice, as seen in the NIELS study [29], in which interruptions of treatment until disappearance of the AEs seemed to be the norm.

AEs have been reported in chronic use of HHIs, especially on the gastrointestinal and musculoskeletal levels [15,35,36]. A post hoc analysis of the sonidegib phase II BOLT study and the expanded-access, open-label vismodegib study revealed that patients treated with sonidegib had a later median time to onset for all AEs than patients treated with vismodegib, except fatigue and weight decrease [34]. After three treatment cycles of vismodegib, the cumulative rates of muscle spasm, dysgeusia, and alopecia were approximately 60%, 60%, and 25%, respectively, while these rates for sonidegib were 32.9%, 15.2%, and 5.1%, respectively [34]. A possible explanation may be the different pharmacokinetic profiles of both HHIs. Sonidegib has a higher volume of distribution (9170 L) [37] than vismodegib (16.4–26.6 L) [38] and a lower plasma level [39,40], suggesting better tissue distribution of sonidegib. In fact, the concentration of sonidegib is six-times higher in skin than in plasma [37], which may explain potential differences in efficacy and toxicity [15].

Although the pivotal trial with sonidegib showed no negative impact on the quality of life in aBCC patients [15,41], in practice, even low-grade AEs due to HHI treatment can generally be expected to affect quality of life in the long term. This can lead to treatment interruption or even discontinuation, which might affect the clinical outcome [14]. There is, therefore, a significant unmet need for new strategies to provide effective long-term treatment of patients with laBCC, which will be discussed here.

## 3. Targeted Treatment with HHIs—How to Balance Efficacy and Toxicity

### 3.1. On-Off Treatment Schedules

Several studies aimed at improving the tolerability of HHIs while maintaining their efficacy. This should reduce the number of patients who discontinue HHI therapy due to AEs. Thus, some studies have sought to determine whether an intermittent dosing regimen leads to a reduction of AEs, and thus to a prolonged duration of treatment (DOT).

One study of distinct dosing regimens was the MIKIE study, a randomized, regimen-controlled, double-blind, phase II trial, which evaluated two intermittent vismodegib dosing regimens in patients with multiple BCCs [20]. A total of 229 patients were randomly assigned (1:1) to treatment group A (150 mg oral vismodegib per day for 12 weeks, then three rounds of eight weeks of placebo daily and 12 weeks of 150 mg vismodegib daily alternately), or treatment group B (150 mg oral vismodegib per day for 24 weeks, then three rounds of eight weeks of placebo daily and eight weeks of 150 mg vismodegib daily alternately). Both arms showed less AEs ≥ grade 3 and a longer treatment duration than the continuous-dose studies STEVIE and ERIVANCE (Table 2). The STEVIE study was a multicenter, open-label trial that enrolled 1215 patients with laBCC or mBCC [42]. The primary endpoint of this study was safety. Patients received 150 mg oral vismodegib per day on a continuous basis in 28-day cycles.

In a retrospective analysis of 48 patients treated with vismodegib, 10 patients received dose adjustment (on-off scheme with 4–16 weeks interruption) to reduce AEs [21]. Seven patients had fewer AEs than before, and three patients stopped treatment because of AEs. Four of the ten patients had a CR, three had a PR (one discontinued), two an SD (one discontinued), and one patient observed progressive disease (PD; one discontinued).

In a retrospective analysis of clinical charts, Tronconi et al. examined the feasibility of tailoring vismodegib administration to toxicity in 17 patients with multiple or locally advanced BCC [22]. Standard practice of the institution allowed on-off therapy (150 mg per day for four weeks with a subsequent break of two weeks) after continuous treatment for two months. In all patients (*n* = 8) on alternate schedules, CR and AEs resolved during the first interruption of therapy. Four patients on a continuous daily dose (*n* = 9) had CR, while five patients progressed; two of the latter patients died after one and three months for disease-correlated reasons, respectively. The median DOT was extended to 27.3 months (range 11.7–38.8 months) in the alternative group, compared to 5.4 months (range 2.4–6.7 months) in the continuous group.

Villani et al. reported a case series of 10 patients with multiple BCCs treated with different vismodegib treatment regimens [23]. Three patients were treated with a regular dosing regimen (150 mg daily), four patients received a modified treatment regimen based on dose adjustment (on-off scheme with eight or twelve weeks interruption) according to the number and severity of reported AEs, and three patients received a prophylactic dose reduction (150 mg every other day) from baseline in order to avoid severe AEs. Patients who received a holiday regimen (during treatment or at baseline) reported a good AE profile characterized by mild adverse events leading to longer treatment durations.

### 3.2. Dose Reduction

Another approach to improve the tolerability of HHIs and the DOT is to reduce the recommended dose of one capsule daily. The reduction to one capsule every other day in case this is required to reduce side effects is specifically within the label of sonidegib [37]. Further dose adjustments have been investigated in various studies.

In a retrospective analysis, 23 patients with laBCC were treated with vismodegib [16]. In total, 16 patients experienced more than one AE during vismodegib treatment; 11 of these patients received a modified treatment scheme based on dose adjustment, and two patients received a prophylactic dose reduction from initiation onward to avoid severe AEs. Dose reduction ranged from once every two days to once every seven days. The overall response appeared comparable to response rates reported for the full dosage. Dose reduction during treatment decreased AE severity in about 55% of patients.

In another retrospective case series, 8 of 21 patients with laBCC were treated with vismodegib 150 mg on Monday to Friday, with a 2-day drug holiday [17]. The remaining 13 patients were initially treated with vismodegib 150 mg daily. Patients in the Monday to Friday group had similar efficacies with a milder adverse effect profile. No severe AEs were observed in the Monday to Friday group, compared with 38% in the group with a daily dosing schedule. In total, 25%, 62.5%, and 12.5% of Monday to Friday patients showed no, mild, and moderate AEs, respectively. By contrast, the daily group experienced 15%, 46%, and 0% of no, mild, and moderate AEs. The initial average time to onset of AEs was 7.7 weeks in the Monday through Friday group, compared with 6.4 weeks in the daily group.

Clinical outcomes were similar in patients treated with an every-other-day dosage of vismodegib (*n* = 8) in a retrospective case series, but mild AEs led to a longer DOT than in patients treated with 150 mg daily [18]. No severe AEs were described, and five out of eight patients had no side effects at all.

In a retrospective case series of 20 sonidegib patients, nine patients received dose adjustment to reduce AEs and prevent discontinuation [19]. Patients were switched to an every-other-day dosage after 12–24 weeks. Patients receiving alternative dosage regimens showed comparable clinical responses, with milder AEs (grade 1–2) than patients receiving daily dosage. CR occurred in six patients and PR in three patients.

In the BOLT study, 16.5% of patients with laBCC or mBCC who were treated with 200 mg QD of sonidegib required a dose reduction due to occurrence of AEs [44]. The ORR did not differ significantly between patients with or without dose reduction and interruption (50.0%; 95% CI 21.1–78.9 vs. 57.4%; 95% CI 43.2–70.8).

As the low-grade toxicities associated with long-term HHI treatment may lead to treatment interruption or discontinuation, management of AEs and an increase in tolerability are important to keep patients on treatment. The management of AEs has been well summarized by Lacouture et al. [14]. On-off treatment schedules and dose-reduction regimens (Table 3) are effective methods to reduce the intensity of common AEs and maintain patients on treatment without losing efficacy.

### 3.3. Re-Challenge after Relapse without Other Intercurrent Treatments

In a retrospective multicenter observational study, 116 patients with laBCC who had CR on vismodegib and subsequently discontinued treatment were studied [46]. The median relapse-free survival (RFS) was 18.4 months (95% CI 13.5–24.8 months). The RFS rate at 36 months was 35.4% (95% CI 22.5–47.9%) for the total population and 40.0% (95% CI 25.7–53.7%) for patients with Gorlin–Goltz syndrome. The median overall survival (OS) was not reached, and the rate at 36 months was 85% (95% CI 74.6–96%). Risk factors associated with RFS were laBCC for limbs and trunk (hazard ratio 2.77; 95% CI 1.23–6.22). A total of 50% of patients who relapsed during follow-up were re-treated with vismodegib, of whom 85% experienced an objective response, 37% a CR, and 48% a PR.

### 3.4. Maintenance of Remission

Once (complete) remission has been achieved, the goal is to maintain this state for as long as possible. Initial studies have investigated the effect of long-term low-dose HHI treatment after remission.

In a single-center retrospective observational study, 42 patients with aBCC who had CR after vismodegib treatment were divided into two groups: 64% of patients received a once-weekly maintenance dose of 150 mg vismodegib for one year after CR, while 36% of patients did not receive a maintenance dose due to more severe AEs [47]. In patients who continued low-dose vismodegib treatment, no BCC recurrence occurred during the 1-year follow-up. In addition, no AEs were reported except for mild dysgeusia in 48% of patients and mild muscle pain in 29.6%. In comparison, 26.6% of patients who discontinued vismodegib treatment experienced a BCC recurrence during the 1-year follow-up. All previously reported AEs had disappeared.

**Figure 2:**
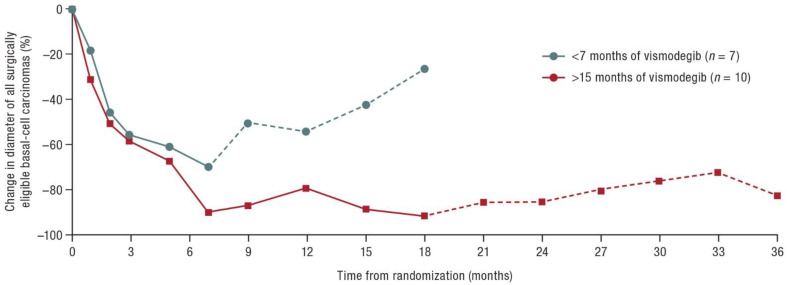
Median percent change in diameter of all surgically eligible BCCs. Solid lines represent time on vismodegib; dashed lines represent time off vismodegib. Modified according to [48].

In a multicenter, randomized, double-blind, placebo-controlled phase II study, 41 patients with BCNS treated with vismodegib or placebo were monitored [48]. In patients taking vismodegib continuously for at least 15 months (*n* = 10), the effect against BCC was maintained (i.e., there was no return to baseline tumor burden) for 18 months after drug discontinuation (Figure 2).

In a retrospective observational study, patients who extended vismodegib for more than two months after CR had higher disease-free survival (DFS) than patients treated for ≤2 months (mDFS 470 vs. 174 days, *p* = 0.008) [49]. The total number of treatment days correlated significantly with the DFS (*p* < 0.0001).

In the currently ongoing SONIBEC trial (NCT04806646), patients with laBCC who achieved CR after HHI therapy are treated with sonidegib on a 14-day on-off regimen. With grade 3 or grade 2 toxicity (except alopecia) lasting more than 28 days, the treatment regimen will be changed to seven days on and 21 days off. The primary endpoint is maintenance of tailored treatment for 12 months after CR.

### 3.5. Resistance Mechanisms of HHIs

As shown previously, vismodegib and sonidegib are effective drugs for the treatment of laBCC. However, some patients may be resistant to treatment, termed intrinsic resistance, or may develop resistance to treatment after the initial response, termed acquired resistance [50]. Numbers of acquired resistance on HHIs are not known from pivotal studies, but the occurrence of intrinsic and acquired resistance was investigated in a subset of 148 patients of the STEVIE study [51]. In this trial 9 patients (6.1%) discontinued treatment because of intrinsic resistance and 14 patients (9.5%) because of acquired resistance.

Regardless of the type of resistance, most patients with HHI-resistant BCC have mutations in *SMO* (Figure 1C) [52,53,54]. Pricl et al. examined the tumors of two vismodegib-resistant patients enrolled in the STEVIE study with intrinsic and acquired mutations, respectively [52]. The patient with intrinsic resistance had a SMO G497W mutation, which is known to impede entry of the drug into the binding site. The patient with acquired resistance had a SMO D473Y mutation known to directly impair the binding affinity of vismodegib. Further analysis of *SMO* mutations present in 50% of BCCs and resistant to SMO inhibitors revealed two distinct mechanisms of resistance: mutations at the binding site (observed in patients with acquired resistance), and mutations that abrogate SMO autoinhibition and induce constitutive SMO activity (observed in patients with intrinsic and acquired resistance) [55].

Tumors with mutations in the canonical HH pathway, typically mutations in *PTCH1* and *SMO*, are often responsive to SMO antagonists [56]. However, tumors with non-canonical activation of the HH pathway, independent of SMO, are less sensitive to SMO inhibition.

One approach to deal with HHI resistance in patients with BCC is to switch agents after intrinsic or acquired resistance becomes apparent with differing success [57,58,59]. Combination therapy, such as itraconazole [60] or the phosphoinositide-3-kinase (PI3K) inhibitor buparlisib [61], might be helpful.

## 4. Immune Checkpoint Blockade with Anti-PD-1-Blocking Antibodies

### 4.1. Efficacy of PD-1 Inhibitors in BCC

An open-label, multicenter, single-arm phase II study resulted in primary analysis data of the PD-1 antibody cemiplimab from patients with laBCC who had developed resistance while on HHI treatment or were intolerant to previous HHI therapy [24]. Reasons for discontinuation of prior HHI therapy were disease progression (71%), intolerance to previous HHI therapy (38%), or no better than SD after nine months on HHI therapy (8%). A total of 84 patients received cemiplimab 350 mg every three weeks for up to 93 weeks or until progression or unacceptable toxicity. The ORR was 31% (95% CI 21–42), of which 6% had CR, 25% had PR, 49% had SD, and 11% showed PD. The Kaplan–Meier estimate of DOR was 85% of responders at 12 months. Grade 3–4 treatment-emerging AEs occurred in 48% of patients and serious treatment-related AEs occurred in 35% of patients. Latest long-term follow-up data up to 40 months revealed a mPFS of 16.5 months (95% CI 8.6–21.4) [62].

There was no clinically meaningful association between objective response and programmed cell death 1 ligand 1 (PD-L1) expression, tumor mutational burden (TMB), or expression of major histocompatibility complex (MHC)-I [24]. The MHC-I expression level on tumor cells was low or absent in some patients with high TMB who did not respond. Exploratory correlative biomarker analyses did not support the use of PD-L1 or TMB to predict the response to (or the clinical benefit of) cemiplimab in laBCC. This study provides the first prospective evidence that MHC-I downregulation is a potential mechanism of immune evasion during anti-PD-1 therapy of high TMB tumors, which could explain the lower ORR and CR in this study compared to similar studies in melanoma and squamous cell carcinoma (SCC). The optimal therapeutic approach in future clinical trials depends on whether reduced MHC-I expression in BCC is reversible for therapeutic advantage and synergistic with immunotherapy.

Another proof-of-principle, non-randomized, open-label study evaluated the efficacy of pembrolizumab (200 mg every three weeks) for patients with aBCC [63]. The ORR was 44% (95% CI 14–79) and the median DOR was 68 weeks (range 31–82 weeks). The authors observed no significant correlation between PD-L1 expression before treatment with pembrolizumab and best percentage change in BCC diameter.

### 4.2. Rationale for the Use of Immune Checkpoint Blockade in BCC

**Figure 3:**
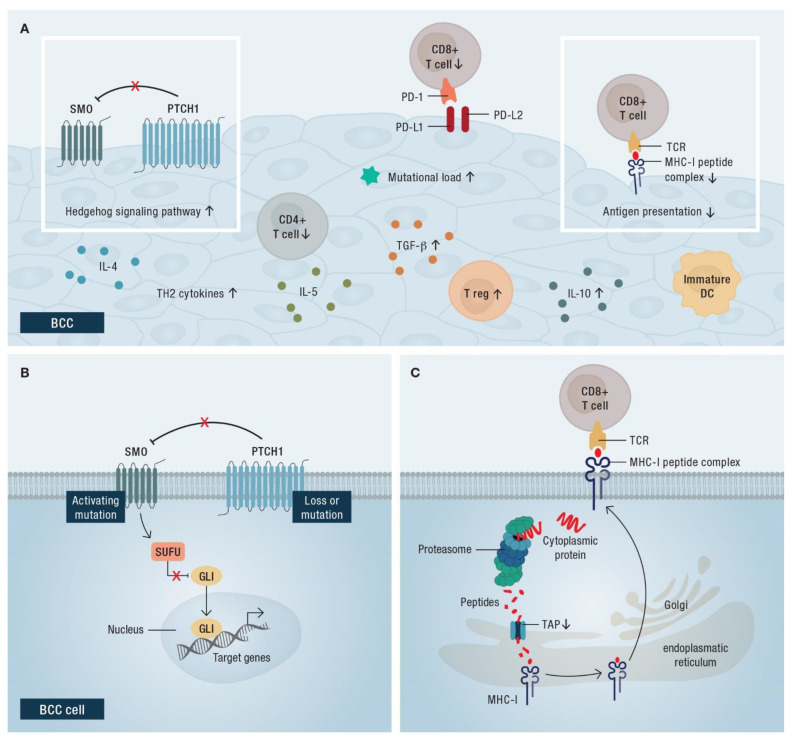
Schematic representation of the immunological aspects of the basal cell carcinoma (BCC). (**A**) The tumor microenvironment is characterized by decreased infiltration of CD4+ and CD8+ T cells and increased presence of regulatory T cells (T reg). Dendritic cells (DC) of an immature phenotype are present in in the tumor microenvironment of laBCC. Increased expression of TH2 cytokines (interleukin [IL]-4, IL-5) and transforming growth factor (TGF)-β, as well as IL-10, supports an immunosuppressive environment surrounding the tumor. In addition to a high mutational load, tumor cells are characterized by the expression of the inhibitory molecules programmed cell death 1 ligand 1 (PD-L1) and PD-L2, which can suppress T cell activity by binding to programmed cell death protein 1 (PD-1) on T cells; (**B**) loss-of-function mutations in *PTCH1* or activating mutations in *SMO* activate the HH signaling pathway in laBCC cells; (**C**) decreased expression of the transporter associated with antigen processing-1 (TAP-1) and major histocompatibility complex (MHC)-I lead to decreased recognition of tumor cells by the immune system.

Since immunotherapy in cutaneous melanoma has proved to be successful, the immunogenicity of non-melanoma skin cancers, such as cutaneous SCC (cSCC), has increasingly become an area of research interest. Pathogenesis occurs via UV-induced DNA damage to epidermal keratinocytes; thereby leading to genetic alterations. Notably, cutaneous neoplasms have the highest TMB of all cancer types (Figure 3A,B) [64]. Accordingly, a high TMB likely causes high expression of neoantigens, leading to high immunogenicity.

As PD-L1 expression levels are associated with response to PD-1 inhibitors in other cancer types, the expression of PD-1 and PD-L1 on BCC tumor cells and intratumoral immune cells was measured in two different studies. One study reported expression of PD-L1 on 22% of the analyzed BCC specimens and on 82% of tumor-infiltrating lymphocytes [65]. The other study found expression of PD-L1 in 50% of treatment-naïve BCCs and on 50% of tumor-infiltrating lymphocytes in these patients [66]. Interestingly, HHI pretreatment seemed to significantly increase the intensity of PD-L1 staining on both tumor cells and tumor-infiltrating lymphocytes.

However, compared to melanoma and cSCC, antigen presentation is reduced in BCC, resulting in poorer immunogenicity. Several immunological aspects of the tumor are responsible for this. Decreased expression of the transporter associated with antigen processing-1 (TAP-1) and MHC-I, which are important for antigen presentation, leads to decreased recognition of tumor cells by the immune system (Figure 3C) [64,67]. BCCs show inconsistent expression of the human leukocyte antigen (HLA)-ABC and an absence of HLA-DR [68]. Furthermore, the presence of immature dendritic cells in the stroma of BCC reduces the visibility of the tumor to the immune system (Figure 3A) [69]. Decreased infiltration of the tumor by CD4+ and CD8+ T cells and the increased presence of regulatory T cells as well as TH2 cytokines (IL [interleukin]-4, IL-5), transforming growth factor (TGF)-β, and IL-10, also lead to an immunosuppressive environment in the tumor (Figure 3A) [64,68,70,71]. Treatment with HHIs leads to upregulation of MHC-I on BCC cells and increased infiltration of CD4+ and CD8+ T cells into the tumor [72].

Blank et al. developed a “cancer immunogram” that illustrates the interactions between cancer and the immune system to help guide treatment choices [73]. This interaction is based on a set of largely independent parameters that can vary widely among patients. The immunogram comprises seven parameter classes. Whether the immune system recognizes the tumor as foreign depends largely on antigen presentation and mutational load. The general immune status provides information about the numerical composition of the immune cells, especially lymphocytes. T cell-based tumor elimination requires infiltration of tumor-reactive T cells into the tumor. This process can be inhibited by deficient T cell priming, a mechanical barrier due to tumor-associated fibrosis, inadequate vascularization of the tumor, or the absence of T cell-attracting chemokines. Furthermore, checkpoints, soluble mediators, or metabolic factors may hamper the activity of the T cells. Finally, it is also necessary that the tumor cells are sensitive to a T cell response. Interdisciplinary detection of these conditions based on the cancer immunogram, both during the cancer-immune interaction and after immunotherapy, may help in therapeutic classification of the individual patient.

## 5. What Follows Immunotherapy?

### 5.1. Re-Challenge with HHI after Immune Checkpoint Blockade

What follows immunotherapy if it is not successful? The authors oversee some hitherto unpublished cases from their clinical practice of patients who were successfully switched from immunotherapy back to HHI treatment. One patient with BCC unsuitable for surgery was initially treated with sonidegib (200 mg QD). Due to PD the patient was switched to pembrolizumab, but as the patient relapsed further under immunotherapy, treatment was changed back to sonidegib 200 mg QD with clinical benefit over several months.

**Figure 4:**
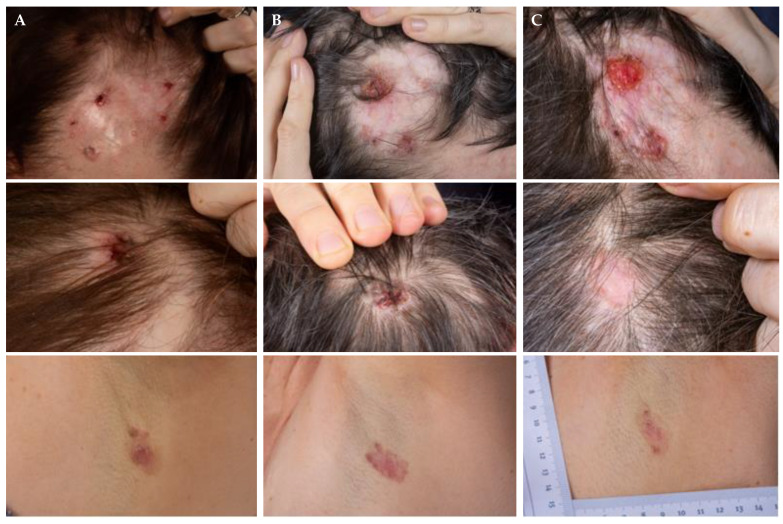
Case history of a patient with Gorlin–Goltz syndrome and multiple BCCs before pembrolizumab treatment (**A**), 7 months after pembrolizumab (at progression) and before sonidegib treatment (**B**), and 5 months after sonidegib treatment (**C**).

Another patient with Gorlin–Goltz syndrome and multiple BCCs received vismodegib with an on-off treatment schedule as initial therapy. This patient was switched to pembrolizumab 200 mg every three weeks due to PD after vismodegib (Figure 4A). After initial SD on immunotherapy, disease progression occurred (Figure 4B). Therefore, treatment was switched back to HHIs. Under treatment with sonidegib 200 mg QD, the patient experienced partial SD as well as PR as a mixed response (Figure 4C).

### 5.2. Combination Therapies

There are a few case reports on the combination of HHIs and itraconazole. Itraconazole is a systemic antifungal agent that has been identified as a potent antagonist of the HH signaling pathway [74]. In an exploratory phase II trial, itraconazole (200–400 mg per day) showed a significant reduction of BCC size in some patients. Itraconazole reduced tumor size and promoted re-epithelialization in BCC tumors of 8 of 29 vismodegib-naïve patients, reducing the tumor area by 24% (95% CI, 18.2% to 30.0%) [75]. Since the binding sites to SMO are at different locations for itraconazole and vismodegib, the two drugs do not interfere with each other and result in a deeper suppression of the pathway [76]. This opens the possibility of a combined therapy which may improve clinical outcome.

One case report describes an elderly man whose disease progressed on both vismodegib as well as on pembrolizumab [60]. The combination of sonidegib 200 mg daily and itraconazole pulsed 100 mg daily for two weeks followed by two weeks off resulted in the disappearance of the intracranial lesion; the intranasal and sinus lesions were largely stable to slightly improved. In two further patients, the combination of vismodegib and itraconazole was given as first-line therapy, resulting in complete healing of the patient during the 16-month follow-up [77].

Ramelyte et al. analyzed two cases of patients with BCC receiving sonidegib in combination with itraconazole following vismodegib and pembrolizumab [78]. These patients achieved disease control with acceptable tolerability.

## 6. Ongoing Studies and Future Developments

### 6.1. Combination of HHI and Immunotherapy

Treatment with HHI upregulates the expression of MHC proteins, which may increase immune visibility of the tumors and enable an immune-related tumor response [79].

The study mentioned above by Chang et al. compared the combination of pembrolizumab (200 mg every three weeks) and vismodegib (150 mg daily; *n* = 7) with pembrolizumab monotherapy (*n* = 9) for patients with aBCC previously progressive on vismodegib [63]. The ORR in the pembrolizumab monotherapy group was 44% (95% CI 14–79). Patients in this group experienced a median DOR of 68 weeks (range 31–82 weeks). In the group treated with pembrolizumab and vismodegib the ORR was 29% (95% CI 4–71) and the patients had a median DOR of 53 weeks (range 28–78 weeks). The response rate of the combination group was not superior to that of the monotherapy group. However, the two groups were not directly compared, and the sample size was limited.

An ongoing investigator-initiated trial (NCT04679480) is addressing the question of whether HHI and PD-1 inhibitors act synergistically in laBCC and achieve better outcomes than anti-PD-1 monotherapy. This prospective, open-label, single-center, phase II study will enroll 20 patients with aBCC. All patients will receive the combination of cemiplimab (350 mg every three weeks) and pulsed sonidegib (200 mg daily for two weeks followed by two weeks off). Pulsed HHI therapy is expected to show a better safety profile with preserved capacity to increase immune visibility of the tumors. Given the different mechanisms of action of the two classes of drugs, the combination of HHI and anti-PD-1 is expected to be synergistic.

### 6.2. Current Research

**Figure 5:**
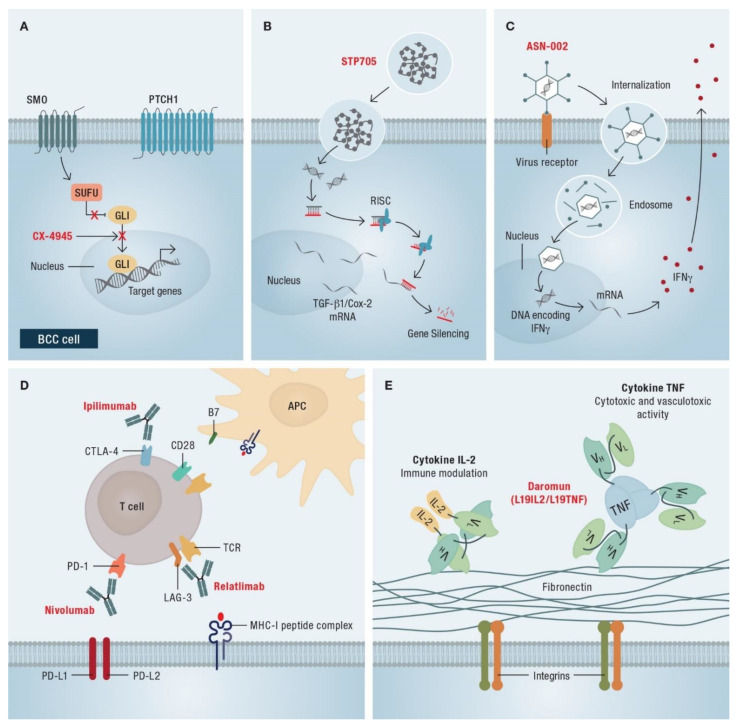
Schematic representation of the modes of action of novel drugs for basal cell carcinoma (BCC) therapy. (**A**) Loss-of-function mutations in *PTCH1* or activating mutations in *SMO* activate the HH signaling pathway in BCC cells. CX-4945 acts on the terminal HH signaling components by inhibiting casein kinase CK2. Thus, the signal transduction pathway is interrupted; (**B**) STP705 consists of two small interfering RNA (siRNA) oligonucleotides packaged with a histidine-lysine (HK) polymer as a delivery system. The siRNA intermediates bind to an RNA-induced silencing complex (RISC), and then selectively degrade the complementary single-stranded target RNA. As the siRNA of STP705 targets the mRNA of transforming growth factor (TGF)-β1 and cyclooxygenase (Cox)-2, respectively, it downregulates the expression of TGF-β1 and COX-2, resulting in tumor growth inhibition; (**C**) ASN-002 functions as a recombinant adenovirus vector, delivering the human interferon (IFN)-γ gene into BCC cells. IFN-γ secretion results in a local IFN-γ concentration within the tumor; (**D**) the checkpoint inhibitor nivolumab blocks binding of programmed cell death protein 1 (PD-1) on T cells to its ligands programmed cell death 1 ligand 1 (PD-L1) and PD-L2 on tumor cells. Relatlimab is an antibody directed against the inhibitory molecule lymphocyte-activation gene (LAG)-3. Binding of cytotoxic T-lymphocyte-associated protein (CTLA)-4 to the B7 molecules as antagonist of CD28 on antigen-presenting cells (APCs) is blocked by ipilimumab. These checkpoint inhibitors may release the T cells from immunosuppressive mechanisms of the tumor microenvironment; (**E**) Daromun (L19IL2/L19TNF) consist of recombinant interleukin (IL)-2 and tumor necrosis factor (TNF)-α cytokines (respectively), fused to a human single-chain variable fragment directed against the extra-domain B (ED-B) of fibronectin (L19). After binding of the L19 moieties to the fibronectin on tumor cells, the cytokines can trigger an immune response against ED-B fibronectin-expressing tumor cells.

Currently, there are several ongoing studies on new therapeutic approaches for BCC. While the HHIs block the onset of HH signal transduction, CX-4945 acts on the terminal HH signaling components by inhibiting casein kinase CK2 (Figure 5A). Thus, CX-4945 would potentially be an alternative in tumors resistant to SMO inhibitors. An ongoing study (NCT03897036) is determining the recommended phase II dose and the schedule for CX-4945 administration in patients with laBCC or mBCC.

STP705 is a novel drug that consists of two small interfering RNA (siRNA) oligonucleotides that target expression of TGF-β1 and cyclooxygenase (Cox)-2 mRNA, respectively (Figure 5B). The siRNA combination will be packaged with a histidine-lysine (HK) polymer as a delivery system. STP705 is expected to downregulate the expression of TGF-β1 and COX-2, resulting in tumor growth inhibition and providing an alternative, non-invasive approach for the treatment of BCC. The efficacy and safety of STP705 is being evaluated in an open-label, dose-escalation study (NCT04669808) in adult patients with trunk or extremity (non-peri-orbital/-anogenital/-facial/-scalp) BCC lesion suitable for excision. 

The efficacy and safety of intralesional ASN-002 in combination with vismodegib will be addressed in another study (NCT04416516). ASN-002 has been designed for intratumoral administration of genes of interest into target cells. It functions as a recombinant adenovirus vector; in this case, delivering the human interferon (IFN)-γ gene into BCC (nodular and superficial) tumors (Figure 5C). As a result, these cells transcribe and translate the IFN-γ-DNA, leading to local IFN-γ concentration within the tumor.

A phase II trial (NCT03521830) evaluates the efficacy of nivolumab, alone or in combination with relatlimab (anti-LAG-3 [lymphocyte-activation gene 3] antibody) or ipilimumab (anti-CTLA-4 [cytotoxic T-lymphocyte-associated protein 4] antibody) in treating patients with unresectable laBCC or mBCC (Figure 5D).

The DUNCAN study (NCT04362722) investigates the efficacy of intratumorally administered daromun (L19IL2/L19TNF) in patients with high-risk, localized injectable lesions of BCC or cSCC. L19IL2 and L19TNF are recombinant IL-2 and tumor necrosis factor (TNF)-α cytokines (respectively), fused to a human single-chain variable fragment directed against the extra-domain B (ED-B) of fibronectin (L19; Figure 5E). After intratumoral injection, the L19 moieties of each immunocytokine bind to the ED-B domain of fibronectin on tumor cells in the tumor neovasculature. The cytokines can then trigger an immune response against ED-B fibronectin-expressing tumor cells.

## 7. Conclusions

The current standard of care of laBCCs that are neither amenable to surgery nor radiation therapy is treatment with HHIs, which show good response rates and DOR. However, toxicities in the course of long-term treatment can lead to a decreased quality of life, and consequently to therapy interruption or even discontinuation. To keep patients on the effective HHI therapy, various strategies are being explored in the clinic to reduce toxicity while maintaining efficacy, such as dose reduction and on-off treatment schedules. After therapy pause and recurrence, a re-challenge with HHI can be performed.

Nevertheless, some patients show primary or acquired resistance to HHI treatment. In these cases, PD-1 inhibitors can be used as second-line therapy. However, they show lower response rates than HHIs and may also be accompanied by significant toxicities. The low response rates to PD-1 inhibitors may be due to the low immunogenicity of BCCs. A key question therefore remains—what follows immunotherapy? The combination of HHIs and itraconazole or PD-1 inhibitors is, therefore, under investigation. In addition, new substances, including relatlimab, and intralesional approaches are being investigated in initial studies. Our understanding of molecular and immunological mechanisms continues to evolve, and we can expect exciting innovations in the treatment of laBCC in the near future.

## Figures and Tables

**Table 1:** Efficacy of sonidegib (18-month analysis) and vismodegib (21-month analysis) in patients with laBCC per RECIST criteria.

Patients with laBCC	Sonidegib 200 mg QD [25] Central View RECIST-like	Vismodegib 150 mg QD [26] Central View RECIST
	*n* = 66	*n* = 63
ORR, *n* (%); 95% CI	40 (60.6); 47.8–72.4	30 (47.6); 35.5–60.6
CR, *n* (%)	14 (21.2)	14 (22.2)
PR, *n* (%)	26 (39.4)	16 (25.4)
SD, *n* (%)	20 (30.3)	22 (34.9)
PD, *n* (%)	1 (1.5)	8 (12.7)
Unknown, *n* (%)	5 (7.6)	3 (4.8)
DOR, median, months	26.1	9.5

RECIST, response evaluation criteria in solid tumors; QD, once daily; ORR, objective response rate; CI, confidence interval; CR, complete response; PR, partial response; SD, stable disease; PD, progressive disease; DOR, duration of response.

**Table 2:** Discontinuation rates in three vismodegib studies using on-off treatment schedules (MIKIE) or continuous dosing (STEVIE, ERIVANCE).

	MIKIE [20] *n* = 229	STEVIE [42] *n* = 1215	ERIVANCE [43] *n* = 104
AEs leading to discontinuation	23% Group A: 23%, Group B: 30%	31%	21%
AE ≥ grade 3	31%	44%	56%
mDOT (weeks)	71	38	56

AE, adverse event; mDOT, median duration of treatment.

**Table 3:** Overview of published alternative dosing regimens of HHIs that showed improved tolerability while maintaining efficacy.

Reference	Study Type	Quality of Evidence *	Number of Patients	HHI	Treatment Schedule
**On-off Treatment**					
Dréno et al. 2017 [20]	RCT, phase II	1	116	vismodegib (150 mg/day)	12 weeks on, 8 weeks off
			113		24 weeks on followed by 8 weeks off, 8 weeks on
Scalvenzi et al. 2019 [21]	retrospective case series	4	10	vismodegib (150 mg/day)	4–16 weeks off
Tronconi et al. 2020 [22]	retrospective case series	4	8	vismodegib (150 mg/day)	4 weeks on, 2 weeks off
Villani et al. 2020 [23]	retrospective case series	4	4	vismodegib (150 mg/day)	8–12 weeks off
**Dose Reduction**					
Woltsche et al. 2019 [16]	retrospective case series	4	13	vismodegib (150 mg/day)	1/2–1/7
Wong et al. 2020 [17]	retrospective case series	4	8	vismodegib (150 mg/day)	Mo–Fr, 2 days off
Villani et al. 2020 [18]	retrospective case series	4	8	vismodegib (150 mg/day)	1/2
Villani et al. 2021 [19]	retrospective case series	4	9	sonidegib (200 mg/day)	after 12–24 weeks 1/1 switch to 1/2

* Rated according to [45]; RCT, randomized controlled trial; 1/2, once every second day; 1/7, once weekly; Mo, Monday; Fr, Friday; 1/1, once daily.
